# A model study of the combined effect of above and below ground plant traits on the ecomorphodynamics of gravel bars

**DOI:** 10.1038/s41598-020-74106-9

**Published:** 2020-10-13

**Authors:** Francesco Caponi, David F. Vetsch, Annunziato Siviglia

**Affiliations:** 1 Laboratory of Hydraulics, Hydrology and Glaciology, ETH Zurich, 8093 Zurich, Switzerland; 2 Department of Civil, Environmental and Mechanical Engineering, University of Trento, 38123 Trento, Italy

**Keywords:** Geomorphology, Freshwater ecology

## Abstract

Both above- and below-ground plant traits are known to modulate feedbacks between vegetation and river morphodynamic processes. However, how they collectively influence vegetation establishment on gravel bars remains less clear. Here we develop a numerical model that couples above- and below-ground vegetation dynamics with hydromorphological processes. The model dynamically links plant growth rate to water table fluctuations and includes plant mortality by uprooting and burial. We considered a realistic hydrological regime and used the model to simulate the coevolution of alternate gravel bars and vegetation that displays trade-offs in investment of above- and below-ground biomass. We found that a balanced plant growth above- and below-ground facilitates vegetation to establish on steady, stable bars, because it allows plants to develop traits that maximise growth performance during low flow periods and thus survival during floods. Regardless of the growth strategy, vegetation could not establish on migrating bars because of large plant loss by uprooting during floods. These findings add on previous studies suggesting that morphodynamic processes play a key role on determining plant trait distributions and highlight the importance of including the dynamics of both above- and below-ground plant traits for predicting shifts between bare and vegetated states in river bars.

## Introduction

Feedbacks between plants and river morphodynamics processes are increasingly recognised to be key in shaping river morphology. By interacting with flow and sediment transport, plants are able to influence the evolution of river channels and bed forms such as river bars, creating preferential sites for vegetation establishment. In turn, hydromorphological processes not only provide essential resources for plant growth, but also control plant survival trough erosion and deposition of the riverbed during floods^1^. In the current climate scenario, where seasonal flow regime is expected to change^2^, it is of utmost importance to predict how riparian vegetation will respond to altered disturbance regimes and to which extent feedbacks between vegetation and river hydromorphology will be affected^3^. However, while the need for quantitative tools able to inform river managers is mounting^4^, basic understanding of these feedbacks remains at an early stage.

Vegetation establishment in rivers is known to depend on the balance between biological and physical factors. Such balance has been generally quantified by comparing vegetation resistance to scours and the morphodynamic potential of floods to rework the riverbed^5,6^. This conceptualisation has also been applied to quantify changes in the biogeomorphic succession^7^ and the windows of opportunity necessary for vegetation recruitment^8^. More recently, plant response to disturbances has been hypothesised to be predicted by specific plant functional traits that express the relationships between environmental factors, ecological processes and a given species^9–11,16^. Trait-based approaches, where plants are grouped depending on their functional traits, have shown to predict well vegetation response to flow regimes^9,12,13^ and changes in biogeomorphic phases^14^. However, a clear understanding on how (functional) vegetation types may influence river ecomorphodynamics remains limited to a handful of studies^15,26^.

Both above- and below-ground plant traits contribute to plant’s ability to interact with hydromorphological processes and cope with specific disturbances^10,16,17^. Rapid growth of rooting depths and root biomass after recruitment can reduce the probability of uprooting by scour^18,11,19,20^, but it is also key for securing constant access to the groundwater, which serves as main water resource for riparian plants^21^. A number of studies has evidenced that plant growth rate varies greatly depending on groundwater dynamics^22,23^. The density and morphology of the plant canopy, which is directly exposed to water flow during floods, is known to determine the effect of plants on flow resistance and on the reduction of sediment transport^24–26^. Plant height, as well as stem biomechanics, represent key traits to account for when predicting plant resistance to sediment burial^15,27^, occurring when flow stage lowers to the base level after floods and sediment is deposited on the riverbed surface^28^. Morphological differences in plant structure may result in trade-offs between the capacity of plants to act as ecosystem engineers and their establishment success. These were found to be key in structuring marsh ecosystems in tidal and coastal environments^29–31^. However, the effect of combinations of different above- and below-ground traits, along with their development in connection with environmental factors, remains poorly explored on fluvial systems.

Despite modelling efforts to include a more detailed description of vegetation-related feedbacks are mounting^32,33^, there is still a lack of appropriate models that links environmental factors with plant morphological traits and describes the associated feedbacks. So far, models have mostly treated vegetation as a whole^34^, not distinguishing between above- and below-ground traits, or have focused on spatial and temporal scales that hindered a clear identification of their role^35,36^. Moreover, model applications tend to simplify the natural variability of the hydrological regime, overlooking the combined effects of floods with different magnitude, duration, and frequency on vegetation survival, and of water table fluctuations during low flow periods on vegetation growth.

**Figure 1:**
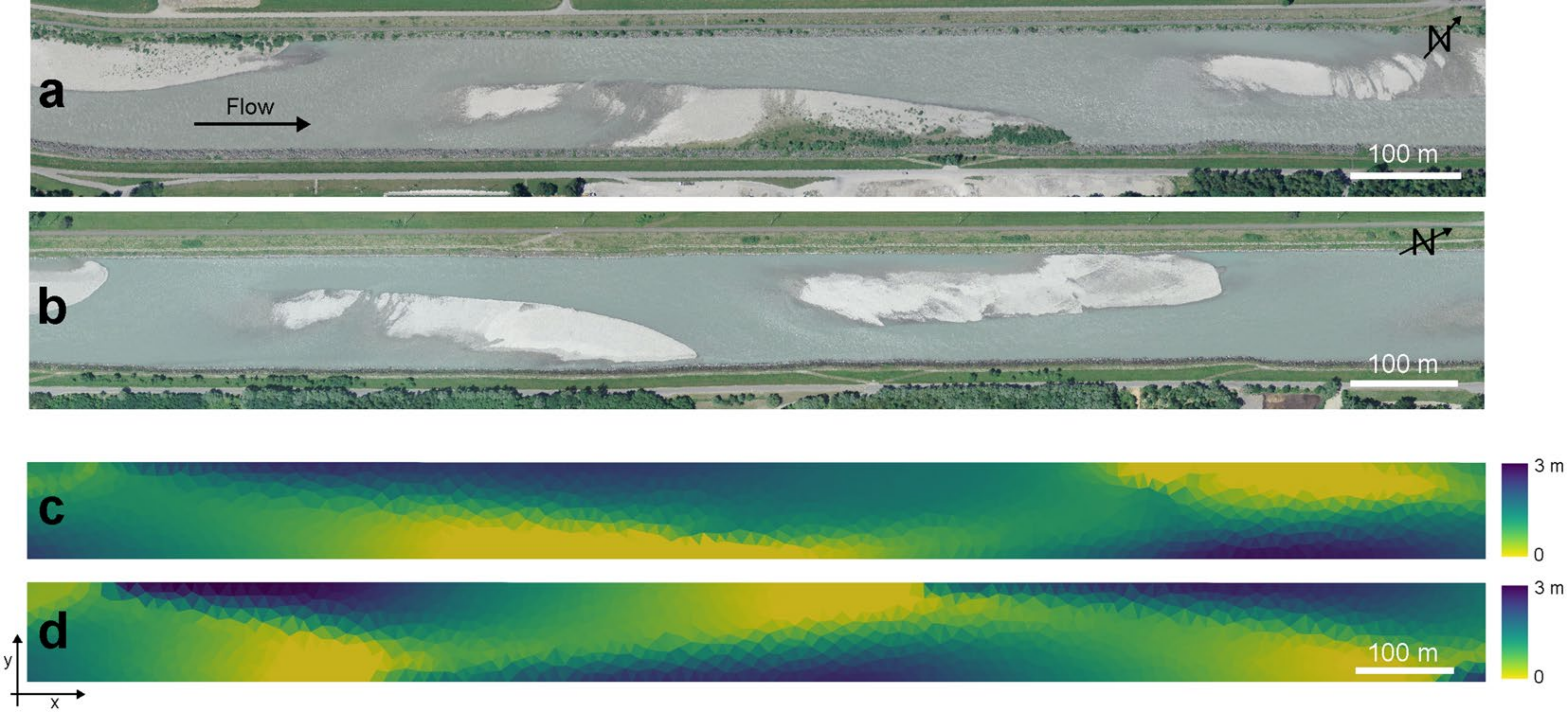
Alternating bar pattern with steady and migrating bars. Aerial images of the Alpine Rhine river (Switzerland-Liechtenstein) taken in 2011 in the reach between Landquart and the Ill river confluence and retrieved from Geodaten ©Swisstopo (https://geovite.ethz.ch) showing: (**a**) steady (partially vegetated) bars and (**b**) migrating (bare bed) bars. Examples of the water depth distribution at discharge of $Q=10$ $\mathrm{m}^3/\mathrm{s}$ generated by the hydromorphological model: (**c**) steady bars and (**d**) migrating bars. These two configurations were used as initial conditions for numerical simulations with vegetation.

The goal of this work is to quantify the role played by both above- and below-ground traits, and their relative evolution, on the ecomorphodynamics of gravel bars. To achieve this goal, we developed a novel ecomorphodynamic model that includes the main feedbacks between above- and below-ground morphological traits and river morphology and takes into account the vegetation growth dynamics as a function of the water table fluctuation during low flow periods. We then performed a series of numerical experiments simulating the co-evolution of alternate gravel bars and vegetation. As model study, we build upon the case of the Alpine Rhine river in Switzerland, which is characterised by an alternate bar morphology with steady and migrating bars (Fig. 1a,b). Steady bars are likely to be stable during floods because of the limited morphodynamic pressure, while migrating bars are subject to erosion and deposition processes that lead to downstream bar migration^37^. Recent observations showed that the area covered by vegetation has been increasing since 2005 only on steady bars, reaching about 25% of the total bar areas after years in which the bars remained mostly bare^38^. This was attributed to particularly favourable hydrological conditions, characterised by sufficient long free-disturbance periods that permitted vegetation to grow and resist periodic removal during floods. Previous studies have also found that vegetation encroachment is likely to be favoured on less disturbed areas such as steady bars because of a reduced morphodynamic pressure^39,40^. In this study, we provide insights on how vegetation types, which differ in the development of above- and below-ground traits, may influence the transition between vegetated and bare states on gravel bars.

## Ecomorphodynamic modelling

Our starting point is a two-dimensional shallow water model that solves the hydromorphodynamic problem by integrating numerically the depth-averaged shallow water equations coupled with the Exner equation, which describes the time evolution of riverbed elevation^41^. When paired with a description of the vegetation dynamics and its effect on flow and sediment transport, such a model was demonstrated to reproduce key ecomorphodynamic processes^34^. In this study, we model feedbacks between hydromorphodynamic processes and vegetation including a description of both above- and below-ground plant traits and their dynamics. In particular, we consider that vegetation impacts flow and sediment transport by modifying the flow resistance, the bed shear stresses, and the threshold for the onset of bed load transport. In turn, morphodynamic processes occurring during flood events are responsible for two main vegetation mortality mechanisms, which are, plant uprooting and sediment burial, while water level fluctuations during low flow periods between floods control the vegetation growth.

### Hydromorphodynamic processes

River hydromorphodynamics is simulated using the numerical model BASEMENT (freeware software)^41^ and the computational domain is discretised using a triangular unstructured grid. First, the model solves the hydrodynamic problem by using the Manning-Strickler approach for evaluating the hydraulic roughness, in which the bottom shear stress, τ, reads as

1 $$\begin{aligned} \mathbf{{\tau }} = \frac{\rho g \mathbf {u}|\mathbf {u}|}{K_s^2h^{1/3}}\quad , \end{aligned}$$

where $\rho$ is the water density, *g* is the gravitational acceleration, $\mathbf {u}$ is the flow velocity vector, *h* the water depth and $K_s$ the Strickler coefficient (the inverse of the Manning coefficient *n*). Second, the sediment continuity equation (Exner equation) is solved to obtain the evolution of the bottom elevation $z_b$ of a riverbed composed of a uniform sediment. The bed load transport intensity is evaluated using the standard Meyer-Peter and Müller formula^42^, as a function of the excess of the Shields shear stress $\theta$ above a threshold value ($\theta _{cr}=0.047$), where

2 $$\begin{aligned} \theta = \frac{|\mathbf{{\tau }}|}{(\rho _s -\rho ) g d_s} \end{aligned}$$

and $\rho _s$ and $d_s$ are the sediment density and diameter, respectively.

### Vegetation description

**Figure 2:**
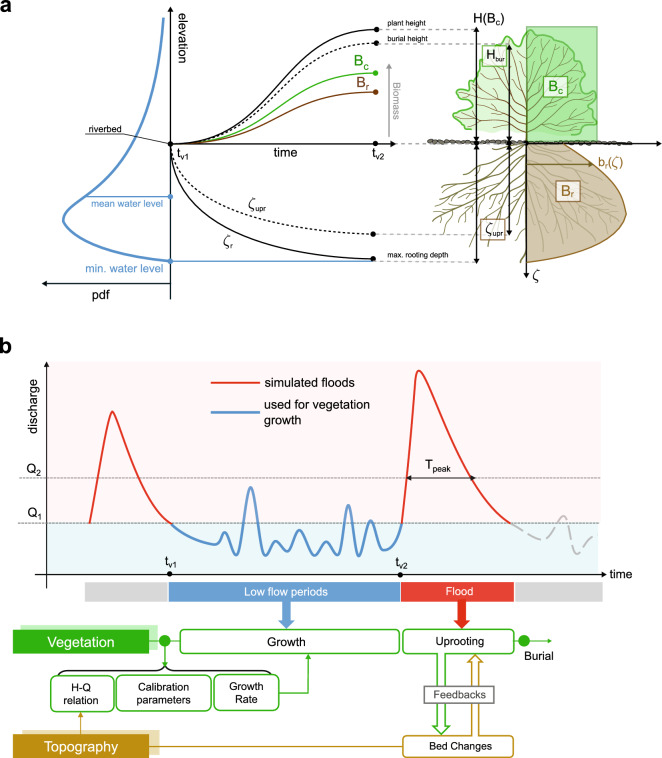
Schematic illustration of the ecomorphodynamic model functioning. (**a**) Vegetation representation in the model includes both above- (canopy) and below-ground (roots) plant traits. Vegetation growth is driven by the water table level fluctuation, which is represented by its probability density function [Eq. (13)] calibrated with water level data during low flow periods (light blue area in the panel). Vegetation in one grid cell can only grow if the bed elevation is greater than the mean water table level, while plant roots cannot deepen below the minimum water table level. (**b**) The model accounts for two distinct phases: (i) low flow periods (between $t_{v,1}$ and $t_{v,2}$), which are periods between two consecutive simulated floods, where vegetation can grow and river is morphodynamic inactive (i.e. $\theta < \theta _{cr}$ in all grid cell); (ii) flood periods, where feebdakcs between vegetation and hydromorphological processes are active. Vegetation can be uprooted during the entire flood event and/or can die because of burial at the end of the flood. Floods are defined from the discharge time series as periods when the discharge is above $Q_2=800$ $\mathrm{m}^3/\mathrm{s}$ for at least $T_{peak}=12$ h. Simulated floods starts at a base discharge $Q_1$ of 500 $\mathrm{m}^3/\mathrm{s}$.

Vegetation is described by a total dimensionless biomass density, *B*, which is partitioned into two components, above-ground, $B_c$ (subscript *c* stands for canopy) and below-ground, $B_r$ (subscript *r* stands for roots) (Fig. 2a). The above-ground biomass is considered to be evenly distributed along the plant height, *H*. Conversely, the below-ground biomass is characterised by a dimensionless vertical density distribution $b_r(\zeta )$ that extends downward in the $\zeta$-direction, from the riverbed surface ($\zeta =0$) to the rooting depth $\zeta _r(t)$, i.e. the maximum depth reached by roots at a generic time, *t* (Fig. 2a). $b_r(\zeta )$ is calculated via the stochastic model developed by Tron et al.^43^. The model assumes that fluctuations of the water table level follow the water level in the channel, which produce an alternating sequence of root growth and decay periods at each riverbed depth $\zeta$. This behaviour is then described as a stochastic process and solved to obtain a steady-state solution for $b_r(\zeta )$ (see Appendix for the formulation^43^), which depends on the mean frequency, magnitude, and decay rate of water table fluctuations and represents a key component in our model, controlling plant growth rate and vegetation resistance to uprooting.

### Vegetation growth dynamics

The fluctuation of the water table level is one of the main driving factors controlling growth performances of riparian plants^22^. The ability of species to rapidly grow roots tracking the water table was found to be key for determining the growth rate of plants and their potential establishment success on bars^44^. Highly variable water table levels during growth periods tend to produce water stress that reduces plant growth because of reduced root respiration, while long free-inundation periods may be similarly harmful for plants that fail to grow roots deep enough to secure a connection with the groundwater. To model this link, we use the function $b_r$ as a proxy for the ability of the plant to tolerate inundations, which depends on the riverbed depth reached by the roots a certain time, and relate that to the plant growth rate. We consider that the total biomass density *B* (above- and below-ground components) grows in time (*t*) following a logistic function:

3 $$\begin{aligned} \frac{dB}{dt} = \sigma _B B \biggl (1-\frac{B}{B_{max}}\biggr ) \quad , \end{aligned}$$

where $B_{max}$ is the maximum biomass value (set to 1 in our model) and $\sigma _B$ is the growth rate assumed to vary depending on the dimensionless root density distribution, $b_r$, as

4 $$\begin{aligned} \sigma _B = \phi _{B} \int _{0}^{1}b_{r}(z)dz \end{aligned}$$

where $\phi _{B} [-]$ is a scaling factor and $\int _{0}^{1}b_{r}(z) dz$ represents the steady-state dimensionless root biomass. With Eq. (4), we assume that vegetation grows faster (higher $\sigma _B$) when plant roots are more developed (at the steady-state). This assumption is largely used in modelling dryland vegetation where plant species act as phreatophytes^45^, similarly to riparian plants. Biomass density decay due to waterlogging is included considering that *B* decreases exponentially with a constant rate of 0.1 when the riverbed level falls below the mean water table level.

We assume that the rooting depth changes over time following an exponential function as

5 $$\begin{aligned} \frac{d\zeta _r}{dt} = \sigma _{r}( \zeta _{r,max}-\zeta _r) \quad , \end{aligned}$$

where $\sigma _r$ is the root deepening rate, which is constant, and $\zeta _{r,max}$ represents the distance between the riverbed surface and the minimum water table level (Fig. 2a). Below such level, riverbed matrix is saturated with pore water and roots cannot grow due to the resulting anoxic conditions^43^. Equation (5) implies that roots grow faster as farther they are from the groundwater and linearly with $\zeta _{r,max}$. This behaviour is representative of phreatophytes plant species that uses groundwater as main source of water and tend to elongate roots to keep pace with the receding rate of the water table level^21,46^.

The proportion of the total biomass growth allocated to each plant component is derived using a mass balance model^47^ by introducing two constant partitioning coefficients, $\lambda _i$ with $i \in (c,r)$ (i.e. canopy and roots). These define the fraction of the total biomass growth allocated above- and below-ground and satisfy $\lambda _c + \lambda _r = 1$ for all times. As a consequence, the growth rates of the two plant components can be written as

6 $$\begin{aligned} \frac{dB_i}{dt} = \lambda _i \frac{dB}{dt} \;. \end{aligned}$$

Here we consider $\lambda _i$ constant, assuming no plasticity in biomass partitioning. The canopy height depends on the above-ground biomass through the function^48^

7 $$\begin{aligned} H(t)=aB_c^b(t) \end{aligned}$$

where parameters *a* and *b* are constant in our model and can be used to modulate the plant height growth.

### Feedbacks between vegetation and hydromorphodynamics

We consider that the above-ground biomass changes the bed roughness by modifying the Strickler coefficient $K_s$, which is used to calculate the flow resistance [Eq. (1)], such as

8 $$\begin{aligned} K_s = K_{s,g}+(K_{s,v}-K_{s,g})\frac{B_{c}(t)}{B_{c,max }} \end{aligned}$$

where $K_{s,g}$ represents the roughness of the bare bed, which depends on the sediment grain size, while $K_{s,v}$ $(<K_{s,g})$ is the roughness of a completely vegetated bed assumed to vary with species-specific canopy characteristics. $B_{c,max}$ is the biomass density that can be achieved when $B=B_{max}$, calculated as $B_{c,max}=\lambda _cB_{max}$. The presence of vegetation is also known to affect the shear stresses acting on the bed surface and responsible for sediment transport. We model the reduction of bottom shear stress by multiplying the total shear stress $\mathbf {\tau }$ by a factor $\gamma <1$ and compute the sediment flux using the reduced Shields stress, $\gamma \theta$^32^. The parameter $\gamma$ ranges between 0 and 1 and it is chosen according to $\gamma =K_{s}(t)/K_{s,g}$, with $K_s$ evaluated by Eq.  (8).

The role of root-enhanced riverbed cohesion on the evolution of a gravel bed river is taken into account increasing the critical Shields parameter when roots are present. This is implemented as^32^

9 $$\begin{aligned} \theta _{cr}=\theta _{cr,g}+(\theta _{cr,v}-\theta _{cr,g})\frac{B_{r}(t)}{B_{r,max}} \end{aligned}$$

in which $\theta _{cr,g}$ and $\theta _{cr,v}$ ($>\theta _{cr,g}$) represent the threshold values for incipient sediment motion on bare and vegetated riverbed, respectively. $B_{r,max}$, as $B_{c,max}$, represents the biomass density that can be achieved when $B=B_{max}$, which is $B_{r,max}=\lambda _rB_{max}$.

Mortality by burial occurs after the end of any flood event if the amount of deposition in a grid cell is higher than a given fraction of the canopy height, i.e. $\Delta z_b > H_{bur}$, where $H_{bur}=\beta _{bur}H$ with $\beta _{bur}\in [0,1]$ (see Fig.  2a) . The parameter $\beta _{bur}$ accounts for the ability of the plant to withstand sediment burial and the reduction of the canopy height due to bending caused by water flow^25^. The uprooting is modelled by defining a critical below-ground biomass $B_{r,cr}$ that has to be excavated by flow erosion until vegetation is uprooted^19^. This is defined as^32^

10 $$\begin{aligned} B_{r,cr}= \int _{0}^{\hat{\zeta }_{upr}}b_r(z)dz = \beta _{upr} \int _{0}^{1}b_r(z)dz \end{aligned}$$

where $\hat{\zeta }_{upr}=\zeta _{upr}/\zeta _r$ represents the ratio between the uprooting depth $\zeta _{upr}$ and the rooting depth $\zeta _r$. $\beta _{upr}$ is a constant parameter that defines the strength of the root system to withstand erosion^32^. Uprooting can occur during the flood event at any time. *B* and $\zeta _r$ are set to their initial values when burial or uprooting occurs.

Riverbed erosion and aggradation processes alter the proportion of above and below-ground biomass during floods. According to the mass balance adopted, vegetation is able to re-allocate biomass at a rate that depends on the partitioning coefficients, $\lambda _i$ [Eq.  (6)]. We consider that buried part of above-ground biomass can convert to roots, while exposed part of below-ground biomass caused by erosion can transform in above-ground biomass. Canopy height is then re-calculated using Eq.  (7) and the rooting depth is adjusted depending on bed level changes. This assumption is justified by the great plasticity observed in riparian species, which are able to easily resprout from buried stems and grow new tissues from exposed roots^49^. When no morphological changes occur, the proportion of biomass allocated above- and below-ground can be calculated as $B_i=\lambda _iB$.

### Model workflow

The model workflow is shown in Fig. 2b and considers an alternating sequence of floods and low flow periods. During each flood event morphological changes occur as a result of the two-way interaction between riparian vegetation and river morphodynamic processes. Vegetation can be uprooted during the entire flood event and/or can die by burial at the end of the falling limb of the discharge. Low flow periods are comprised between two consecutive flood events and may last from months to years. In this phase the riverbed is inactive ($\theta < \theta _{cr}$) and vegetation is allowed to grow and develop above- and below-ground traits.

As a first step, we simulate vegetation growth during a low flow period, given a vegetation cover and a riverbed topography. The biomass growth rate [Eq. (4)] is dynamically computed through the evaluation of the function $b_r$ [see Eq.  (11) in the Appendix], which varies in space depending on the local (cell-wise) variability of the water table level during the growth period. Here we assume that the water table level changes locally with the water surface elevation. This assumption holds true for gravel, uniform substrates where hydraulic conductivity is high^43^. Discharge variability during low flow periods is responsible for changes in water surface elevations, and thus in water table levels. To evaluate the water surface elevation associated with a certain discharge, we derive a water level-discharge h-Q relation for each computational cell by running one hydrodynamic (fixed bed) simulation for a series of discharges. When the water surface elevation is zero, namely when the cell becomes dry at a certain discharge, we assume that the water table level can be calculated by interpolating the water surface elevations of the nearest wet cells. This allow us to transform discharges in water table level time series and to obtain the frequency distribution of water table levels for each cell during low flow periods. The h-Q relation is then used to calibrate the parameters $\lambda _w$, $\eta _w$, $\gamma _w$ required to compute the function ${b}_r$ [see Eqs. (12) and (13) in the Appendix for details] and calculate the growth rate, $\sigma _B$. By numerically integrating Eqs. (3) and (5) for the duration of the specific growth period, we update the vegetation variables and the associated feedbacks. This procedure introduces a link between plant growth rate and low flow regime characteristics, which in turn influences plant traits.

As a second step, we simulate the riverbed evolution during floods, where feedback mechanisms between vegetation and hydromorphological processes are activated. In particular, the uprooting mechanism [Eq. (10)], the correction of the bed shear stress and flow resistance [Eq. (8)], and the critical Shield parameter [Eq. (9)] are updated every time step. After the flood, we used the new river bed topography for updating the vegetation cover including mortality by burial. The resulting vegetation cover and plant traits are then used as initial conditions for the subsequent growth period.

## Numerical simulations

We performed numerical simulations in a channel that resembles the averaged conditions of the Alpine Rhine river in the reach between Landquart and the Ill river confluence in Switzerland^38^, which displays both steady (Fig.  1a) and migrating bars (Fig.  1b). The computational domain is 12 km long, with a width of 85 m, slope of 0.0029 m/m, and mean grain size of 60 mm. The numerical grid is composed by 19,010 triangular cells, which have a mean area of about 60 $\mathrm{m}^2$.

To reproduce a pattern of alternating steady and migrating bars^50^, we included an obstacle attached to the sidewall occupying half of the channel width, placed 2 km downstream the inlet and first run the hydromorphodynamic model with a constant discharge of $Q=1000$ $\mathrm{m}^3/s$ and sediment input (at equilibrium with the boundary cells) over a flat bed channel until the complete formation of alternate bars. The obtained configuration was characterised by three steady bars downstream the obstacle and a series of migrating bars (of about seven) that self-form in the downstream part of the domain. We then run the model with unsteady discharges simulating a series of floods with different magnitude and duration, selected from the major flood events recorded at the gauging station at Bangs along the Alpine Rhine river in the period 1996–2005 (Supplementary Figure S1). The key morphological features, such as height and length, of the resulting steady (Fig.  1c) and migrating bars (Fig.  1d) compared well with the ones measured along the Alpine Rhine river^37,38^. The topography obtained in this way was then used as initial condition to perform model runs including vegetation.

**Figure 3:**
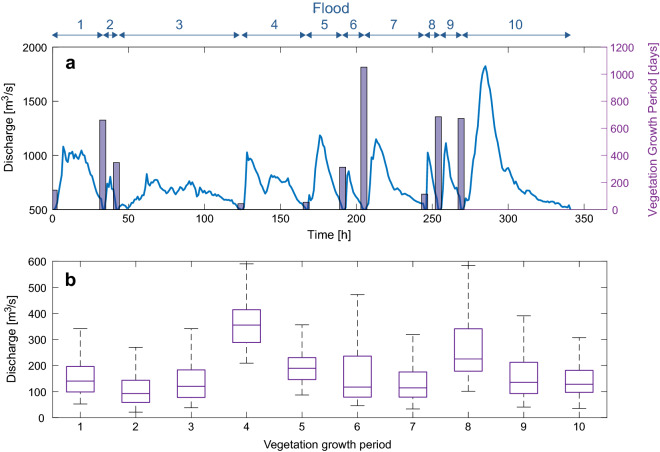
Flood hydrographs used in the numerical simulations and statistic of discharges during vegetation growth periods. (**a**) Flood events are numbered from 1 to 10. Vertical bars indicate the duration in days of the flood inter-arrival periods, which correspond to the vegetation growth periods. (**b**) Box plot of discharges. The period before flood 7 was the longest (> 1000 days), with a mean discharge value slightly above 100 $\mathrm{m}^3/s$. Low values of the mean discharge result in low mean water levels during growth periods, which maximise the area available for vegetation colonisation.

We conducted numerical experiments with vegetation following the workflow presented in Fig. 2b. To qualitatively compare numerical results with field observations, we set up the model using the hydrological conditions (hourly discharge records) of the Alpine Rhine river in the period 2005–2017 (Supplementary Figure S1). In such period, observations show a transition from bare to (partially) vegetated condition on steady bars but not on migrating bars^38^, which we expect to reproduce with the model. To this end, we selected all flood events with discharges above $Q_2=800$ $\mathrm{m}^3/s$ having a minimum duration of $T_{peak}=12$ h (Fig. 2b), which corresponds to events during which significant morphological changes are expected^37,38^. For reducing the computational effort required by the numerical simulations, all the selected floods were cut at 500 $\mathrm{m}^3/s$, assuming that the sediment transport is negligible below this value^37,38^. In this way, we obtained 10 flood events with varying peak discharge and duration (Fig.  3a). We used the recorded discharges between every two consecutive floods as input to the vegetation growth model. Figure 3b reports the statistics of discharges including the first vegetation growth period, which corresponds to the 6 months of records before flood 1. The length of vegetation growth periods corresponds to the number of days between two consecutive floods and they do not include seasonality (see Supplementary Table S1). Therefore, vegetation growth was not paused during winter months.

**Table 1:** Model parameters used for defining vegetation types. $\sigma _r$ is the rooting depth growth rate [Eq. (5)], $\lambda _c$ and $\lambda _r$ correspond to relative investments between above- and below-ground plant structures, respectively [Eq. (6)].

Vegetation type description	Run	$\lambda _c$ $[-]$	$\lambda _r$ [–]	$\sigma _r$ $[1/\mathrm{m}]$
Equal investments in above- and below-ground biomass	AB	0.5	0.5	0.012
More investments in above-ground biomass	AA	0.8	0.2	0.006
More investments in below-ground biomass	BB	0.2	0.8	0.024

We considered three vegetation types with growth strategies that vary according to investment in above- and below-ground traits (Table 1). These included plants that (i) equally invest in above- and below-ground biomass with moderate rate of root deepening (runs AB), (ii) invest mostly in above-ground biomass and slowly deepen the root system (small $\sigma _r$) (runs AA), and (iii) invest mostly in below-ground biomass with a fast deepening rate of roots (large $\sigma _r$) (runs BB). With this choice, we aimed at exploring the effect of vegetation types on modulating feedbacks to morphodynamic processes (i.e. bed friction and root-enhanced riverbed cohesion), mortality mechanisms (burial and uprooting), and plant growth rate. These do not necessarily correspond to species-specific strategies, but can be rather interpreted as functional adaptations to specific environmental conditions^16,51^. Vegetation in runs AA and BB are characterised by specialised growth strategies, which favour the development of either above-ground or below-ground traits, whereas vegetation in run AB adopts a generalist growth strategy.

The bar ecomorphodynamics was quantified through the analysis of vegetation cover, mortality rates by uprooting and burial, and the development of plant traits throughout the whole simulations. Vegetation cover was calculated as the ratio between the number of computational cells with vegetation and the number of cells exposed at a low discharge ($Q=10$ $\mathrm{m}^3/\mathrm{s}$), which represents the maximum bar area that can be colonised by vegetation. The mortality rates were computed by the corresponding cover removed by uprooting or burial after each flood. Plant traits were reported as averages computed over all cells with vegetation. To quantify the morphodynamic potential of the floods tested, we computed the frequency distribution of bed level changes over vegetated areas caused by each of the flood events. This allowed us to also compare the effect of vegetation types on erosion and deposition processes.

## Results

### Steady bars

**Figure 4:**
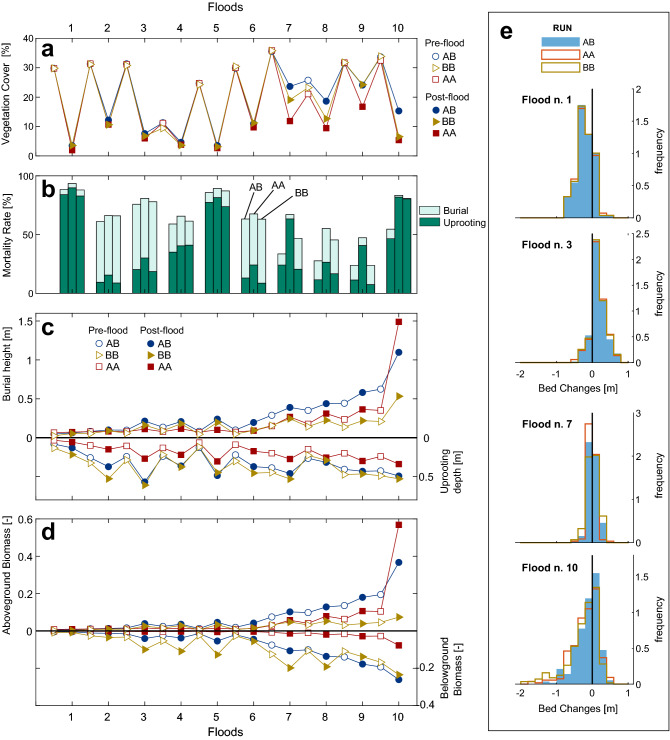
Numerical simulation results for steady bars. (**a**) Vegetation cover. (**b**) Mortality rates by uprooting and burial in % of the pre-flood vegetation cover. (**c**) Burial height (upper panel) and uprooting depth (lower panel). (**d**) Above- and below-ground biomass density. (**e**) Frequency distribution of net bed changes ($\Delta z$) for the three vegetation types after floods 1, 3, 7, and 10.

Figure 4 shows the results of numerical simulations for the steady bars. The area covered by vegetation varied between about 15% in run AB (flood 10) and 0.075% in runs AA and BB (flood 1) displaying an increasing trend after flood 6 (see post-flood cover in Fig. 4a). On average, vegetation occupied about 30% of the bar area before each of the floods, with no significant differences among runs. However, the high water levels registered during the low flow periods before floods 4 and 8 (Fig. 3b) significantly reduced the pre-flood vegetation cover to about 10% and 23% (21% for run AA and 25% for run AB), respectively. Vegetation removal by uprooting and mortality by plant burial significantly reduced the vegetation cover. Mortality rates were above 60 % until flood 6 for all runs, while they reduced in floods 7, 8 and 9 in a different manner depending on the run (Fig. 4b). The reduction of the mortality rates in floods 7, 8 and 9 was more pronounced in runs AB and BB. Uprooting represented the main mortality mechanism during the most severe floods 1, 5 and 10 (all characterised by Q$_{peak}>1100 ~\mathrm{m}^3/\mathrm{s}$), while mortality by plant burial was more pronounced for the moderate floods 2, 3 and 6 (all Q$_{peak}<850~\mathrm{m}^3/\mathrm{s}$). Coupling the information about the mortality rate with the analysis of the frequency distributions of the riverbed changes (Fig. 4e) revealed that floods producing more deposition (e.g., flood 3) increased mortality by burial, while floods producing more erosion (e.g., flood 1) increased uprooting. Above- and below-ground plant traits were not well developed until flood 7 for all runs (Fig. 4c,d). Only the uprooting depths markedly differed among runs already after the first flood, with larger values found in run BB and the smallest in run AA (Fig. 4c). A significant growth of the burial height (Fig. 4c) and the above-ground vegetation density (Fig. 4d) occurred after flood 6. This was due to the long vegetation growth period occurring between floods 6 and 7 (see Fig. 3a). Vegetation in run AB developed taller and denser canopy compared to vegetation in other runs, constantly increasing after flood 7. We observed an increase in mean values of uprooting depths, burial heights, and biomass after each flood, indicating that plants surviving the floods were, on average, the ones with more developed traits. This can be clearly observed comparing pre- and post-flood plant traits for flood 10 (Fig. 4c,d). The uprooting depths (Fig.  4c) were generally longer in run BB than in the other runs, while they were shorter in run AA. They overall showed an increasing trend with time in all runs, but markedly reduced before floods 4 and 8 as a consequence of the high minimum water discharge (level), below which roots cannot elongate. In all runs, the biomass densities, which have a direct effect on morphodynamic processes, did not affect bed level changes until flood 10. This is evident when comparing the bed frequency distributions for floods 1 to 7 (Fig. 4e), where areas subject to scour and deposition did not show any significant differences among runs. However, the areas in which erosion occurred decreased in run AB when compared to the other runs in flood 10, with a corresponding increase in areas displaying sediment deposition. The greatest survival of vegetation in run AB than vegetation in runs AA and BB was not correlated by the uprooting depths (Fig.  4d) but it rather can be attributed to the reduction of erosion due to presence of denser above-ground vegetation (Fig. 4c,e).

### Migrating bars

**Figure 5:**
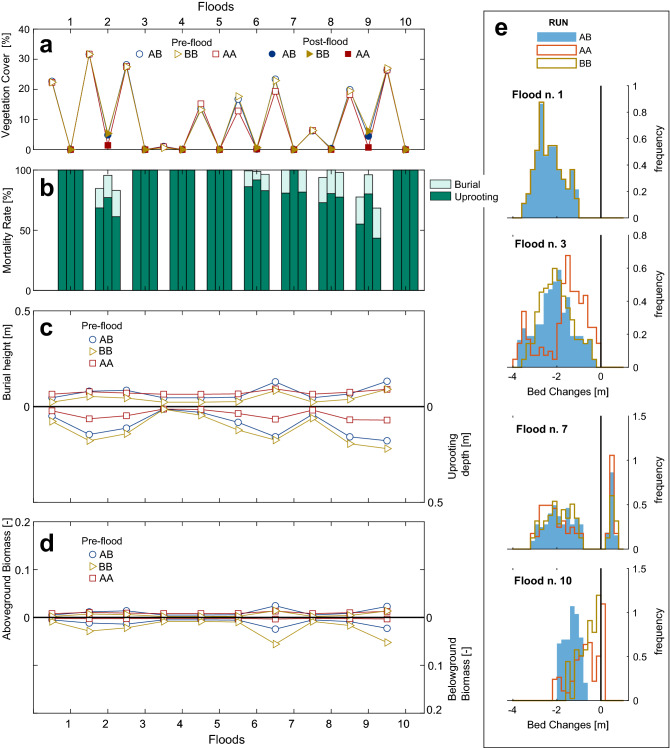
Numerical simulation results for migrating bars. (**a**) Vegetation cover. (**b**) Mortality rates by uprooting and burial in % of the pre-flood vegetation cover. (**c**) Burial height (upper panel) and uprooting depth (lower panel). (**d**) Above- and below-ground biomass density. (**e**) Frequency distribution of net bed level changes ($\Delta z$) for the three vegetation types after floods 1, 3, 7, and 10.

Figure 5 shows the results of the numerical simulations for migrating bars. In all runs, vegetation was completely removed during each of the floods, limiting its expansion on the bars (Fig. 5a). Pre-flood vegetation cover varied widely throughout the simulations, ranging from almost zero (0.005%) before flood 4–32% before flood 2 (Fig.  5a). These variations are controlled by fluctuations in the mean water levels during low flow periods (Fig. 3). The bar area covered by vegetation is generally lower than the cover observed on steady bars for the same period (Fig. 4), because of the different bar height. Only minor differences were found in vegetation cover among runs. Almost the entire area covered by vegetation before each of the floods was removed by uprooting, with only minor contribution of burial (Fig. 5b). Due to the complete removal of vegetation occurring during every flood, plants had no possibility to develop strong resistance (Fig.  5c,d). The frequency distributions of bed level changes show that floods produced erosion that reached a maximum value of 4 m (e.g. flood 1) and no deposition, with the only exception of flood 7 (Fig. 5e). This is a consequence of downstream bar migration, which occurred in all ten floods regardless of the peak discharge. The distributions in floods 3 and 10 varied depending on the run, but it did not display any significant correlation with plant traits. This deviation can be due to the influence of upstream steady bars on the formation of alternate migrating bars and of the resulting location of vegetated patches along the channel.

## Discussion

Above- and below-ground plant traits are important for determining both plant’s response to disturbances such as scour and sediment deposition and its effect on river morphodynamics. Depending on the growth strategy, plants may develop traits specifically adapted to resist, and interact with, specific disturbances but not others. Our results indicate that the effect of different growth strategies (i.e. vegetation types) can be significant for river bar ecomorphodynamics and varies depending on the steady and migrating nature of bars. Along migrating bars, where bar migration causes bed erosion that largely exceeds the anchoring resistance of the roots (i.e. uprooting depths, Fig. 5 and Supplementary Figure S3), plants did not survive to any flood independently from the vegetation type considered. Even low to moderate floods (e.g. flood 3) were able to completely remove pioneer vegetation by uprooting and reset the system to bare riverbed conditions. The tested flood frequencies did not allow plant traits to develop enough for slowing bar migration, which is key for establishment success of plants in alternating bar configurations^38,40^. On the contrary, vegetation was able to colonise more stable (steady) bars, to an extent that depended on the vegetation type. Our results indicate that plants able to develop both above- and below-ground (run AB) were more suited to survive floods and to occupy larger proportion of bar surface than plants with more specialised growth strategies (runs AA and BB, Fig. 4). The larger colonisation area of vegetation in run AB (15% of the bar area) than in other runs (7%) is the combined result of greater above-ground biomass growth, which enabled plants to develop taller canopies and resist burial more efficiently, and the elongation of roots, which guarantees constant access to moist soil boosting overall plant growth and resistance to uprooting (see Fig.  4b,d). Rapid root growth was found to be especially crucial during long low flow periods with low water table level. In such periods (e.g., before flood 7, Fig.  3), vegetation that expresses a trade-offs between above- and below-ground traits were more vulnerable to disturbances. In run AA, plant roots failed to reach deep, moist riverbeds, which both limited plant growth rate and resistance to uprooting, whereas vegetation in run BB, despite the fast root growth rate, did not grow enough canopy to build up resistance to sediment burial (see results for flood 7 in Fig. 4). The feedback between root depth and plant growth rate has been previously documented^22^ and found to be important for determining biogeomorphic trajectories^23^. However, it is often not included in numerical models, where low flow regimes are de-coupled to vegetation growth. In this context, our results provide evidence on its importance when modelling ecomorphodynamic processes.

A number of studies have suggested that species distribution can be predicted by the interaction between disturbance regime and key plant traits. Experiments carried out by Perona and Crouzy^12^ (but see also recent modelling studies^20,52^) showed that the vegetation survival to flood can be predicted by the rooting depth distribution prior the flood, as trait responsible to control plant resistance to scour. Floods would tend to remove individuals with less developed root systems, more vulnerable to uprooting, therefore selecting only individuals having longer uprooting depths. This behaviour is captured by our model and can be observed in our results looking at the increase of the mean uprooting depth after each flood event (particularly clear in flood 10, Fig. 4). Recent observations on *Populus nigra* indicate that plants tend to develop short seedlings with flexible stems in order to minimise the effect of the drag and therefore increasing the probability to withstand uprooting in areas subject to frequent erosion processes^17,53^. Based on the results presented, we argue that the strategy of plants that would maximise survival probability in environments that tend to produce scour, such as in migrating bars, would be different from the one that would develop in more stable riverbeds such as steady bars, where both scour and deposition are equally important. In addition, vegetation type was found to be key on steady bars, where more generalist growth strategy was favoured (i.e. run AB), but it was irrelevant along migrating bars, where the high morphodynamic potential of floods muted the effects of vegetation with contrasting growth strategies. A similar behaviour was experimentally observed in sand bars for species exhibiting different above-ground morphology (e.g. tamarisks vs. cottonwood), which were shown to respond similarly to hydromorphodynamic processes in association with high magnitude of erosion rates^18,15,26^.

Factors controlling occurrence and rates of vegetation encroachment in river channels have been long debated. The transition between bare and vegetated riverbeds has been attributed to changes in the balance between vegetation strength and channel mobility induced by relevant discharges^6^. Such balance can vary following a reduction in frequency and magnitude of floods, but also by biotic factors such as the arrival of invasive species with better survival ability and colonisation strategies than native species^54^. Previous modelling studies on alternate bars have shown the role played by flow variability and vegetation characteristics in modulating the transition between vegetated and bare states^33,34,55^, indicating the presence of two stable alternative states on alternate bars, steady-vegetated bars and migrating bare bars. Our results add on these findings, and provide insights on the role played by both above- and below-ground plant traits and their development, as well on the whole flow regime including low flows and floods with different magnitude, duration, and frequency, which are often not considered. Our results suggest that stable bars, which produce rather limited scour and deposition (see Fig.  4 and Supplementary Figure S2), are not necessarily vegetated and that this depends on how traits related to plant resistance to both uprooting and burial develop over time when compared to the flood magnitude and frequency. Vegetation mortality is often included as a threshold mechanism depending on the magnitude of scour (or its proxy) during floods. However, mortality by burial is of utmost importance, as it may account for more than 50% of total vegetated area loss during floods on steady bars, which we found after long lasting, low magnitude floods (i.e. flood 3, Fig. 3). This shows that the prediction of the state transition of bars with stable morphology is more sensitive to variations of flood characteristics and plant trade-offs than of bars where erosion processes dominate (migrating bars).

The time required by plants to establish on bars and activate biogeomorphic feedbacks also depends on specific plant traits. Our findings can be interpreted in the context of the biogeomorphic succession^56^, which consists in distinct phases where the strength of the interaction between plants and morphodynamic processes changes. After seed dispersal, the co-evolution between vegetation and river morphology is dictated by geomorphic disturbances, which limit plant establishment causing uprooting and burial. The transition into a biogeomorphic state, where feedback mechanisms between plants, flow and sediment transport became important for predicting river trajectories, depends on a number of factors, but recent studies have related such transition to species-specific plant traits^17^. The results on steady bars presented here suggest the presence of three phases in which the relative effect of plant traits varies. In a first phase, which can last up to a few years, the growth strategy of plant species may only marginally affect vegetation establishment on bars (Fig. 4 from flood 1 to 6). We found that, when plants can grow enough to develop traits that can counteract the morphodynamic pressure, the vegetation mortality during floods depends on specific traits (i.e. uprooting depth and burial canopy height). However, in this phase, plants were found to not significantly change morphodynamic processes (see bed changes distribution in Fig. 4). Only when plants develop a significant amount of above-ground biomass needed to influence the flow, the system may enter a biogeomorphic feedback state. We argue that reaching this point within the succession is strictly dependent on how vegetation develops traits in time and in response to disturbances, and therefore to the specific trade-offs that emerge from it. This means that the time required by plants to reach this phase (i.e. biogeomorphic feedback window^57^) may be dependent on functional plant traits, as previously suggested^17^. In particular, our findings indicate that plants balancing growth both above and below-ground on steady bars could have shorter biogeomorphic feedback windows than species with more specialised growth strategies.

In this study, we provide a quantitative insight and a modelling framework to investigate the effect of above and below-ground plant traits and their feedbacks on gravel bar ecomorphodynamics. Despite a point-to-point comparison with observations was not in our scopes, our model results agree, on a qualitative level, with recent field observations in the Alpine Rhine river in Switzerland^38^. Results on steady bars showed a gradual increase in vegetation cover on bars, especially after flood 6. Such event corresponds to the flood occurred in 2009 in the Alpine Rhine, where a marked increase in vegetation cover was observed in response to a long free-disturbance period^38^. In agreement with observations, numerical simulations also showed that vegetation colonisation on migrating bars is limited by bar migration causing extensive plant removal. However, a detailed comparison with observation should account for other factors as well. For instance, our simulations did not account for seasonality in vegetation growth. Because of that, we overestimated the growth during periods including winter months (see Supplementary Table S1). In addition, we did not consider variability in dispersal windows across years and assumed that plants can colonise bare substrates every year. However, the model could be used as exploratory tool to shed light on the relationship between river hydromorphology and plant traits, especially important in conjunction with alteration of the frequency and magnitude of floods during vegetation growth periods^3^.

## Supplementary information

Supplementary Information.

## Data Availability

The numerical model BASEMENT is freely available for download at https://basement.ethz.ch/. The datasets generated and analysed during the current study are available from the corresponding author on reasonable request.

## Appendix

### Plant root model

According to the model for calculating the vertical root density distribution^43^, $b_r$ depends on the temporal characteristics of water table oscillations. $b_r$ is defined with respect to a dimensional value that depends on riverbed characteristics, plant species, and other biophysical parameters controlling the amount of roots that can possibly grow within a riverbed matrix. With reference to a downward-oriented vertical axis $\zeta$, with zero at the riverbed surface (see Fig.  2a), the steady-state solution of $b_r$ reads as

11 $$\begin{aligned} {b}_{r}(\zeta ) = \frac{2\sigma (\zeta ) k(\zeta )}{\sigma (\zeta )+\sigma (\zeta )k(\zeta )+1-k(\zeta )}, \end{aligned}$$

with

12 $$\begin{aligned} k(\zeta ) = {\left\{ \begin{array}{ll} \frac{\biggl [ \Gamma \biggl (\dfrac{\lambda _w}{\eta _w},\dfrac{\zeta _1-\zeta -L}{\alpha _w}\biggr )-\Gamma \biggl (\dfrac{\lambda _w}{\eta _w},\dfrac{\zeta _1-\zeta }{\alpha _w}\biggr ) \biggr ]}{\Gamma \biggl (\dfrac{\lambda _w}{\eta _w}\biggr )} \quad &{} \text {if } -\infty<\zeta<\zeta _1-L \\ \frac{1-\Gamma \biggl (\dfrac{\lambda _w}{\eta _w},\dfrac{\zeta _1-\zeta }{\alpha _w}\biggr )}{\Gamma \biggl (\dfrac{\lambda _w}{\eta _w}\biggr )} \quad &{} \text {if } \zeta _1-L<\zeta <\zeta _1 \end{array}\right. } \end{aligned}$$

in which $\Gamma (\cdot$ $,\cdot )$ is the incomplete Gamma function, *L* is the vertical extension of the optimal zone for root growth (capillary fringe), $\zeta _1$ is the lower limit of such zone (below which roots cannot grow), and $\sigma (\zeta )$ corresponds to the ratio between grow and decay rates of roots. Here we consider that $\sigma =1$ for $\zeta \in [0,\zeta _r(t)]$ at any time, *t*. $\lambda _w$, $\eta _w$ and $\alpha _w$ are model parameters, which describe the mean rate ($\lambda _w$), the mean depth ($\alpha _w$) of the water pulses, and the rate of water level decrease in time ($\eta _w$). The water level $\zeta _w$ (defined as a Poisson noise) can be described by its probability density function^43^ (see Fig. 2a) as

13 $$\begin{aligned} p(\zeta _w) = \frac{\alpha _w^{-\lambda _w/\eta _w}}{\Gamma (\frac{\lambda _w}{\eta _w})}e^{\frac{\zeta _w-1}{\alpha _w}}(1-\zeta _w)^{\frac{\lambda _w}{\eta _w}-1} \end{aligned}$$

The parameters $\lambda _w$, $\eta _w$ and $\alpha _w$ are spatially variable in this study and change depending on the river topography and the discharge time series. To derive these parameters, we calibrated the function $p(\zeta _w)$ against the frequency distribution of the water level at a specific location.

### Model parameters

For calculating the root distribution $b_r$ we used $L=1$ m and $\zeta _1=0.8$ m [Eq. (12)]. $\sigma$ in Eq.  (11) is set to 1. For the vegetation model, we adopted $\phi =0.1$ in Eq. (4), while for the mortality mechanisms we set $\beta _{upr}=0.8$ and $\beta _{bur}=0.8$. The maximum increase in the Strickler coefficient was $K_{s,v}=10$ $\mathrm{m}^{1/3}\mathrm{s}^{-1}$ and the maximum increase in the critical Shield parameter $\theta _{cr,v}=0.2$. To integrate the logistic function [Eq. (3)], we used a initial value of $B=0.01$ and $\zeta _r=0.05$ m in Eq. (5). Other parameters used were reported in the main text.
