# Supplementary material for: A model study of the combined effect of above and below ground plant traits on the ecomorphodynamics of gravel bars
Source: Sci Rep. 2020 Oct 13;10:17062. doi: 10.1038/s41598-020-74106-9 (PMC7555904; doi:10.1038/s41598-020-74106-9)
Supplement: Supplementary file 1 — Supplementary Information. [file 41598_2020_74106_MOESM1_ESM.pdf]

Supplementary Information
A model study of the combined effect of above- and below-ground plant traits on the ecomorphodynamics of gravel bars

Francesco Caponi, David F. Vetsch, and Annunziato Siviglia

This documents contains Figures S1, S2, S3, and Table S1.

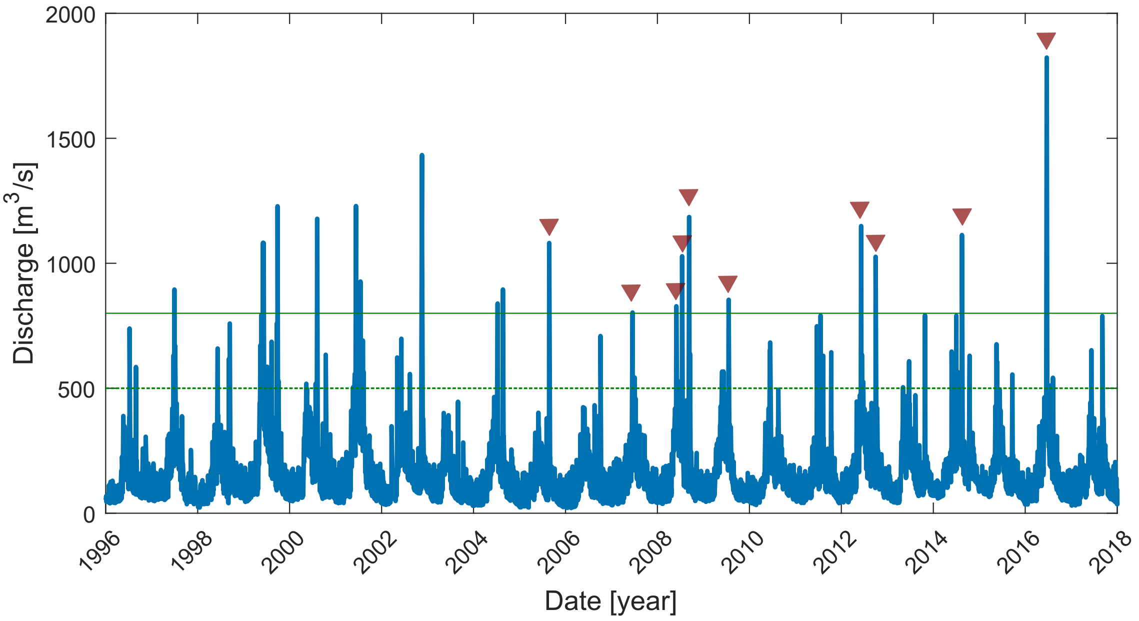

Figure S1: Discharge measurements recorded between 1996 and 2017 at the gauging station in Bangs (Austria). The floods selected for the numerical simulations with vegetation are indicated with a red triangle. The initial alternate bar configuration was obtained using the discharge data from 1996 and 2005.

Number	Growth period		Flood		
	start	end	start	end	duration [hours]
1	01.04.2005	22.08.2005	22.08.2005	24.08.2005	30
2	24.08.2005	15.06.2007	15.06.2007	16.06.2007	6
3	16.06.2007	28.05.2008	28.05.2008	31.05.2008	79
4	31.05.2008	13.07.2008	13.07.2008	15.07.2008	40
5	15.07.2008	07.09.2008	07.09.2008	08.09.2008	21
6	08.09.2008	18.07.2009	18.07.2009	18.07.2009	11
7	18.07.2009	04.06.2012	04.06.2012	05.06.2012	37
8	05.06.2012	27.09.2012	27.09.2012	27.09.2012	6
9	27.09.2012	13.08.2014	13.08.2014	14.08.2014	12
10	14.08.2014	16.06.2016	16.06.2016	19.06.2016	69

Table S1: Start and end dates of the growth periods and flood events used to perform simulations with vegetation, based on the discharge measurements in Bangs (Alpine Rhine river, see Figure S1).

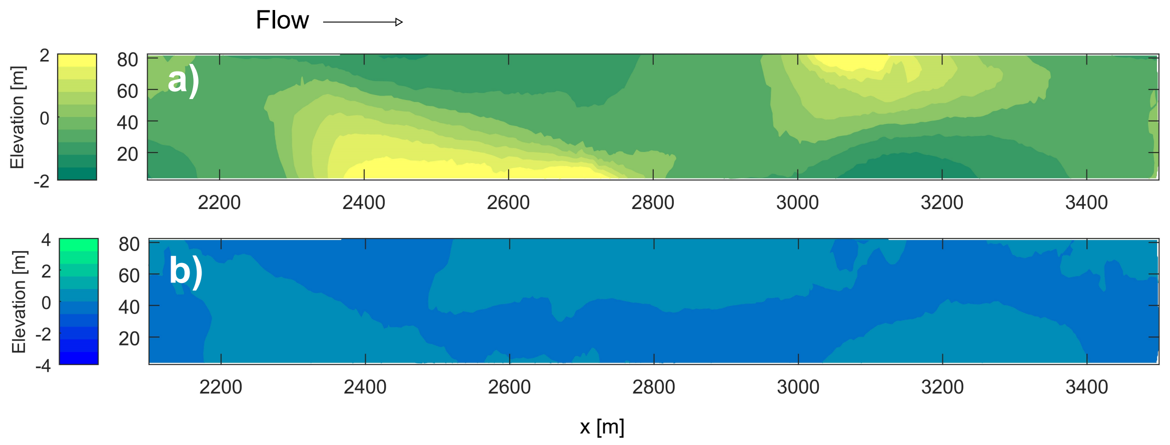

Figure S2: Results of numerical simulations showing a plan-view of the pre-flood bed topography (a) and post-flood bed level changes (b) for steady bars. In (a) elevations are considered with respect to the water level calculated at $Q = 10\ m^3/s$ (i.e. bed elevation above 0 represents the dry bar surface). In (b) elevations refer to the bed level changes with respect to pre-flood bed topography (i.e. scour if elevations are below 0 and deposition is above 0). Results refer to flood 7 for run AB.

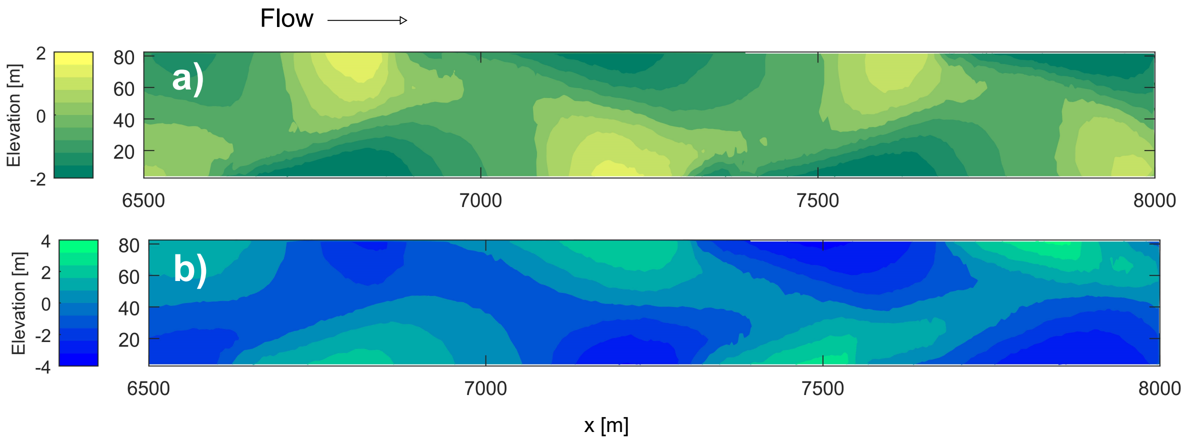

Figure S3: Results of numerical simulations showing a a plan-view of the pre-flood bed topography (a) and post-flood bed level changes (b) for migrating bars. In (a) elevations are considered with respect to the water level calculated at $Q = 10\ m^3/s$ (i.e. bed elevation above 0 represents the dry bar surface). In (b) elevations refer to the bed level changes with respect to pre-flood bed topography (i.e. scour if elevations are below 0 and deposition is above 0). Results refer to flood 7 for run AB.
